# Supplementary material for: Mitochondrial Control Region Database of Hungarian Fallow Deer (Dama dama) Populations for Forensic Use
Source: Animals (Basel). 2024 Jun 28;14(13):1911. doi: 10.3390/ani14131911 (PMC11240637; doi:10.3390/ani14131911)
Supplement: Supplementary file 1 [file animals-14-01911-s001.zip › animals-3061817-supplementary.pdf]

Table S1. Fallow deer hunting seasons in Hungary by age groups and sex. The green cells represent the permission to hunt for the given age and sex group.

	Jan	Feb	Mar	Apr	May	Jun	Jul	Aug	Sep	Oct	Nov	Dec
**Fallow deer buck**												
**Mature buck**												
**2-year-old buck**												
**Doe**												
**Fawn**												
**Calf (both sexes)**												

Table S2. Mitochondrial hypervariable I. and II. region comparison of the number of polymorphic sites based on the reference sequence (NC_020700). Sequences examined came from a previous study [9] and GenBank sequences (GenBank: OR220344-89) on introduced fallow deer populations.

	Hypervariable region I (HVI)																																																		Overall	Average
**Position**	15400	15410	15420	15430	15440	15450	15460	15470	15480	15490	15500	15510	15520	15530	15540	15550	15560	15570	15580	15590	15600	15610	15620	15630	15640	15650	15660	15670	15680	15690	15700	15710	15720	15730	15740	15750	15760	15770	15780	15790	15800	15810	15820	15830	15840	15850	15860	15870	15880	15890		
**Polymorphic sites per 10 base pairs**	0	0	0	1	0	0	1	0	1	0	0	1	2	4	0	3	2	4	4	0	1	0	2	3	0	0	1	5	4	1	6	3	3	2	9	1	2	1	1	0	1	2	5	5	3	1	1	2	2	4	94	1.88
**Coverage (number of sequences)**	13	15	15	18	18	18	380	426	426	426	426	815	815	815	856	856	856	856	856	856	856	856	856	856	856	856	856	856	856	856	856	856	856	856	856	856	856	856	856	859	859	859	859	859	859	470	470	470	470	468		671

	Hypervariable region II (HVII)																																																		Overall	Average
**Position**	15900	15910	15920	15930	15940	15950	15960	15970	15980	15990	16000	16010	16020	16030	16040	16050	16060	16070	16080	16090	16100	16110	16120	16130	16140	16150	16160	16170	16180	16190	16200	16210	16220	16230	16240	16250	16260	16270	16280	16290	16300	16310	16320	16330								
**Polymorphic sites per 10 base pairs**	1	2	2	3	1	2	2	0	0	2	0	0	0	0	0	0	1	0	0	0	0	0	1	1	1	0	0	3	11	4	7	6	4	11	0	0	0	0	0	0	0	0	0	0							65	1.48
**Coverage (number of sequences)**	468	465	465	465	465	465	465	465	465	419	378	378	378	378	378	378	378	378	375	375	375	375	375	375	375	375	375	375	375	375	375	13	13	13	13	13	13	13	13	13	13	13	13	13								288

Table S3. The number of fallow deer haplotypes generated with DnaSP software [26] based on the examination of the HVI or the combined HVI and HVII regions.

	**Reference**	**Hypervariable region I (HVI)**	**Hypervariable region I + II (HVI + HVII)**
**Number of fallow deer haplotypes (k)**	Baker et al. 2017 [9] (730 base pairs)	**25**	**34**
	GenBank: OR220344-89 (510 base pairs)	**37**	**46**

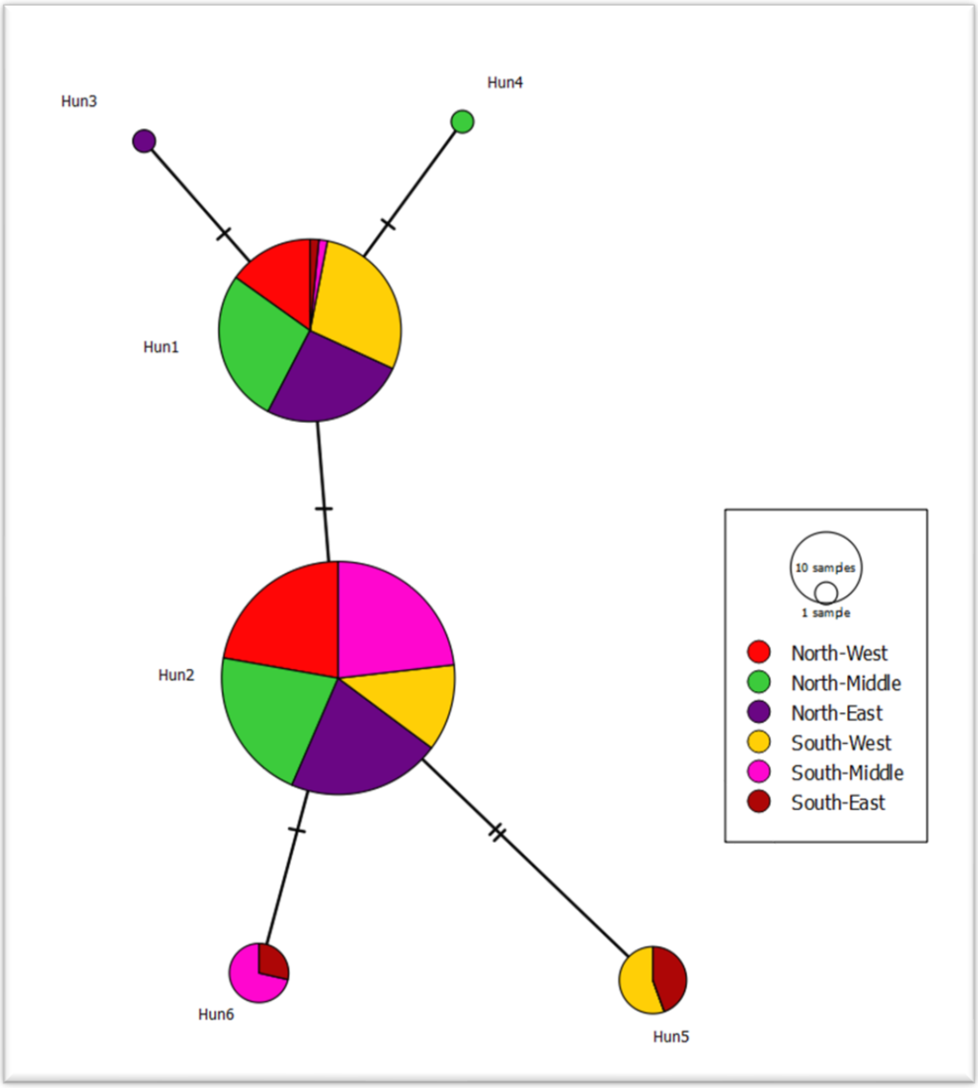

Figure S1. A haplotype network graph was generated with PopART software [47] using the Median Joining Network (epsilon=0). Other network types, such as Minimum Spanning Network, Integer NJ Network, and TCS Network gave the same results. Based on this image, Hun3 and Hun4 derive from Hun1, while Hun5 and Hun6 derive from Hun2. Except for Hun3 and Hun4, which were only observed in one animal, all other haplotypes were detected in multiple populations.

References

9. Baker, K.; Gray, H.; Ramovs, V.; Mertzanidou, D.; Akın Pekşen, Ç.; Bilgin, C.C.; Sykes, N.; Hoelzel, A. Strong population structure in a species manipulated by humans since the Neolithic: The European fallow deer (Dama dama dama). Heredity 2017, 119, 16–26.
26. Rozas, J.; Ferrer-Mata, A.; Sánchez-DelBarrio, J.C.; Guirao-Rico, S.; Librado, P.; Ramos-Onsins, S.E.; Sánchez-Gracia, A. DnaSP 6: DNA sequence polymorphism analysis of large data sets. Mol. Biol. Evol. 2017, 34, 3299–3302.
47. Leigh JW, Bryant D, Nakagawa S (2015) POPART: full-feature software for haplotype network construction. Methods in Ecology & Evolution 6, 1110–1116.
